# Associations of intracranial arterial stenosis and cerebral small vessel diseases with acute ischemic lesions in spontaneous intracerebral hemorrhage

**DOI:** 10.3389/fneur.2026.1810507

**Published:** 2026-05-08

**Authors:** Yutong Hou, Shuna Yang, Yue Li, Wenli Hu, Lei Yang

**Affiliations:** Department of Neurology, Beijing Chao-Yang Hospital, Capital Medical University, Beijing, China

**Keywords:** acute ischemic lesions, cerebral small vessel disease, diffusion-weighted imaging lesions, intracranial arterial stenosis, spontaneous intracerebral hemorrhage

## Abstract

**Background and purpose:**

Cerebral small vessel disease (CSVD) has been declared to diffusion-weighted imaging (DWI) lesions in patients with intracerebral hemorrhage (ICH). The impact of large vessel stenosis on stroke remains uncertain. Therefore, this study examines the relationship between CSVD, intracranial arterial stenosis (ICAS), and DWI lesions in patients with ICH.

**Method:**

A consecutive cohort of 541 patients with spontaneous ICH who had brain MRI and MRA within 14 days of ICH onset was collected and analyzed retrospectively. DWI lesions as well as CSVD were assessed in MRI, and the severity of ICAS was evaluated in MRA. We compared the demographic and clinical features, laboratory parameters, and imaging characteristics of patients with and without DWI lesions and explored the associations of burdens of CSVD and ICAS with DWI lesions in ICH patients using multivariate logistic regression models.

**Results:**

Of the 541 patients enrolled, 137 (25.3%) presented with DWI lesions. Severe total CSVD burden was significantly associated with DWI lesions (OR 3.56, 95% CI 2.22–5.72, *p* < 0.001), and a six-point modified CSVD score (OR 4.66, 95% CI 2.86–7.61, *p* < 0.001) enhanced the prediction of DWI lesions in patients with ICH. Moderate to severe ICAS (OR 2.27, 95% CI 1.39–3.77, *p* = 0.019) was associated with DWI lesions, which became more significant when moderate to severe ICAS coexisted with moderate total CSVD burden (OR 4.44, 95% CI 1.85–10.69, *p* = 0.001). Nevertheless, moderate ICAS without CSVD was not linked to DWI lesions in ICH patients.

**Conclusion:**

Patients with severe CSVD burden are associated with DWI lesions in ICH patients, with a significant correlation also observed between ICAS and DWI lesions. The probability of DWI lesions increases when both ICAS and CSVD are present, suggesting a potential novel association for the development of these lesions.

## Introduction

Acute intracerebral hemorrhage (ICH) can trigger distant cerebral ischemic injury, visible as diffusion-weighted imaging lesions (DWI) in up to 11–46% of patients (1–14). The pathophysiology is multifactorial, but underlying cerebral vasculopathy is likely a key predisposing factor. We hypothesized that the confluence of two distinct vasculopathies, including cerebral small vessel disease (CSVD) and intracranial atherosclerosis (ICAS), would create a high-risk environment, significantly increasing the propensity for DWI lesions after ICH.

Previous studies have found significant links between DWI lesions and individual CSVD markers, such as cerebral microbleeds (CMBs), white matter hyperintensities (WMHs), and enlarged perivascular spaces (EPVSs) (4, 9, 10, 15). However, the results between individual CSVD categories and DWI lesions remain debated. The total CSVD burden score, which incorporates four CSVD markers, can reflect the overall disease impact and may be more effective in examining the relationship between CSVD and DWI lesions. A modified CSVD score that stratifies WMH and EPVS has been proposed, showing better predictive value for intracranial hemorrhage in ischemic stroke patients and good prognosis in ICH patients (16, 17). The investigation of the relationship between the modified total CSVD score and DWI remains limited.

ICAS is a common condition worldwide, sharing risk factors with CSVD such as hypertension and age. Although ICAS and CSVD are different vascular diseases, many studies show they often occur together in people with cerebrovascular symptoms. This simultaneous presence significantly affects the prognosis of patients with ischemic regions (18). Furthermore, ICAS coexisting with CSVD has a stronger link to ischemic stroke. It can predict new stroke and bleeding events in patients with ischemic regions (19). However, most previous studies have focused on patients with ischemic stroke, while research on ICAS in ICH patients remains limited and requires further investigation.

Therefore, this study aims to examine the relationship between the original and modified total CSVD scores and DWI lesions, as well as to analyze the connection between CSVD combined with ICAS and DWI lesions in the ICH population.

## Materials and methods

### Study population

We conducted a retrospective review of data from consecutive patients admitted with acute non-traumatic ICH at Beijing Chao Yang Hospital between June 2013 and July 2022. Primary ICH patients were eligible for our study if they had a baseline computed tomography (CT) scan, brain MRI, and magnetic resonance angiography (MRA) within 14 days of symptom onset. Patients with the following conditions were excluded: (1) isolated intraventricular hemorrhage or subarachnoid hemorrhage; (2) surgical evacuation of hematoma; (3) secondary causes of ICH, such as hemorrhagic transformation of ischemic stroke, aneurysms, cavernomas, arteriovenous malformations, central venous thrombosis, trauma-related causes, or tumors; (4) incomplete sequences for assessing ICAS, CSVD, or DWI lesions, and poor-quality imaging data.

### Clinical data collection

Baseline demographic (age, gender) and clinical data (body mass index, smoking, alcohol), medical histories (hypertension, diabetes, coronary artery disease, ICH, ischemic stroke), and medication use (antiplatelet or antihypertensive) were recorded. Moreover, initial evaluation parameters, including systolic blood pressure (SBP), diastolic blood pressure (DBP), mean arterial pressure (MAP), National Institute of Health Stroke Scale (NIHSS), and Glasgow Coma Scale (GCS) scores on admission, were also collected with well-trained neurologists and noted for analysis. Blood samples were drawn after overnight fasting, the next morning after admission, and the data of white blood cell count, hemoglobin, cholesterol, low-density lipoprotein cholesterol, high-density lipoprotein cholesterol, aspartate aminotransferase, alanine transaminase, fasting glucose, creatinine, urea, fibrinogen, international normalized ratio, and D-dimer were analyzed and recorded.

### Imaging analysis

MRI acquisition was performed using a single 3.0-T MRI scanner, which included the following sequences: T1-weighted imaging, T2-weighted imaging, fluid-attenuated inversion recovery (FLAIR), diffusion-weighted imaging (DWI), apparent diffusion coefficient (ADC), and susceptibility-weighted imaging (SWI). All imaging readers were blinded to each other and to the patients' information.

### Assessment of ICH and DWI lesions

A CT scan assessed hematoma volume and location on admission, checking for ventricular or subarachnoid extension. Hematoma volume was calculated using the ABC/2 method (20). The distribution was categorized into lobes, deep structures, brain stem, and cerebellum. DWI lesions were hyperintense areas under 20 mm with low ADC signal. Restricted diffusion near the hematoma (< 20 mm) was excluded.

### Assessment of CSVD

The imaging markers of CSVDs, including lacune, WMH, EPVS, CMB, and brain atrophy, are evaluated according to the Standards for Reporting Vascular Changes on Neuroimaging (STRIVE) criteria (21).

The lacunes are fluid-filled cavities of presumed vascular origin, hyperintense on T2-weighted and FLAIR images but hypointense on T1-weighted imaging, with a diameter of 3–15 mm. WMH was defined as a signal abnormality in white matter showing hypointensity on T1-weighted images and hyperintensity on T2-weighted and FLAIR images. Periventricular WMH and deep WMH were graded using the Fazekas score (16). WMH with a total Fazekas score of 3–6 was classified as moderate to severe WMH. EPVS were defined as round or linear CSF-isointense lesions, measuring ≤ 3 mm. We graded the EPVS in the basal ganglia (BG-EPVS) and centrum semiovale (CSO-EPVS) using a 5-point visual rating scale (0, no EPVS; 1, 1–10 EPVS; 2, 11–20 EPVS; 3, 21–40 EPVS; 4, over 40 EPVS). Severe EPVS is defined as a score greater than 2. CMBs were defined as rounded or circular spots with low signal intensity on the SWI sequence, measuring up to 10 mm in diameter. The number of CMB, lacunar, and brain atrophy was recorded.

The total CSVD burden score (range 0–4) was calculated by summing one point each for the presence of: (1) lacunes; (2) CMBs; (3) BG-EPVS >10; and (4) Periventricular WMH with a Fazekas score of 3 and/or deep WMHs with a Fazekas score of 2–3 (22).

The modified CSVD burden score (range 0–6) assigns 1 point for the presence of lacunes, 1–4 CMBs, >20 BG-EPVS, moderate WMH (Fazekas grade 3–4); and 2 points for ≥5 CMBs and severe WMH (Fazekas grade 5–6) (17).

### Assessment of ICAS

ICAS was evaluated at the site of the most severe stenosis observed on MRA. Moderate to severe ICAS was defined as 50%−99% stenosis according to the Warfarin-Aspirin Symptomatic Intracranial Disease (WASID) trial criteria (23) or occlusion. ICAS was defined as one of the following arteries: the internal carotid artery (ICA), middle cerebral artery (MCA), anterior cerebral artery (ACA), posterior cerebral artery (PCA), intracranial segment of the vertebral artery, or basilar artery.

Trained physicians (Yue Li, Shuna Yang), who were blinded to clinical data, rated lacunes, EPVS, WMH, CMB, brain atrophy, and ICAS independently. An interobserver reliability test was conducted with 50 subjects, with a 1 month interval between the first and second readings. Kappa values for intra-rater agreement ranged from 0.83 to 0.90. Disagreements were resolved by the third investigator (Wei Qin, neuroradiologist).

## Statistical analysis

Statistical analysis was performed using the SPSS 24.0 statistical package (SPSS Inc., Chicago, IL, USA) and Prism 9. Categorical variables are presented as numbers and percentages, and continuous variables are presented as means and standard deviations, non-normal distributed variables as medians and interquartile ranges. The differences in clinical information, CSVD burden, and ICAS were analyzed between groups with and without DWI lesions. One-way analysis of variance and Kruskal–Wallis test were used for continuous data, and chi-square test or Fisher's exact test for categorical data. The relationships between the presence of DWI lesions and CSVD burden and severity of ICAS were evaluated using binary logistic regression analysis with DWI lesions as a determinant and CSVD burden and ICAS (EPVS, WMH, CMB, lacunar, brain atrophy, total CSVD burden, modified CSVD burden, number and severity of ICAS, and coexistence of CSVD and ICAS) as outcome variables. We constructed three models to adjust for potential confounding factors. Model 1 was adjusted for age and sex and model 2 further adjustments for BMI, ICH volume, NIHSS and other relevant vascular risk factors. Model 3 equaled model 2 with additional adjustments for the presence of ICAS or total CSVD score. The result was presented as odds ratio (OR) and 95% confidence interval (CI). Statistical significance was established at *P* < 0.05.

## Result

**Figure 1:**
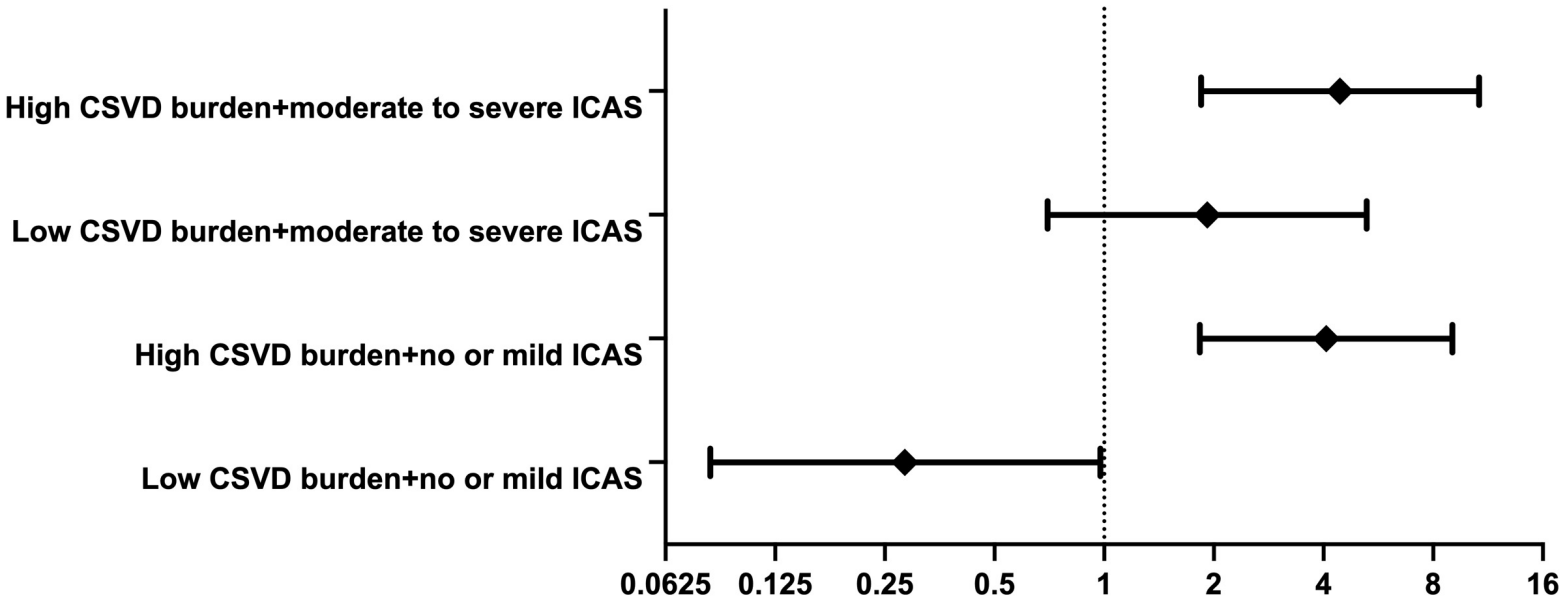
Flowchart of participants according to inclusion and exclusion criteria.

Out of 937 individuals enrolled in our study, 541 met the inclusion criteria, and the flow chart of patient selection is shown in Figure 1. Among these patients, the mean age was 58.0 ± 14.9 years, and 392 (72.5%) were male. The median baseline hematoma volume was 8.0 cm3, and the median (interquartile range) GCS score was 15 (14, 15), with a median NIHSS score of 2 (0, 6). Thirty-one percent of bleeds were lobar, 60.7% deep, 5.5% involved the brainstem, and 5.4% involved the cerebellum.

Among all the included ICH patients, 137 (25.3%) presented with DWI lesions, and 14 patients had more than one lesion, which were symptomatic. DWI lesions were mainly located in the cortical and subcortical regions of the lobes and scattered across multiple vascular territories. Eighty-four (61.0%) patients had DWI lesions ipsilateral to the hematoma, 45 (33.0%) had lesions contralateral to the hematoma, and 8 (6.0%) had bilateral lesions, as shown in the Supplementary Figure S1. Tables 1, 2 show the clinical and imaging features of all patients and subgroups with and without DWI lesions.

**Table 1:** Demographic, clinical and laboratory features between subjects with and without DWI lesions.

Variables	All	DWI(+)	DWI(-)	*p*
	***n*** = **541**	***n*** = **137**	***n*** = **404**	
Age, year	58.0 ± 14.9	62.6 ± 14.4	56.2 ± 14.6	< 0.001
Sex, male, *n* (%)	392 (72.5)	104 (75.9)	288 (71.3)	0.295
BMI, kg/m^2^	25.3 (22.7, 27.5)	25.4 (22.0, 27.5)	25.3 (22.8, 27.5)	0.653
Medical history, *n* (%)				
Hypertension	326 (60.3)	90 (65.7)	236 (58.4)	0.133
Diabetes	110 (20.3)	39 (28.5)	71 (17.6)	0.006
CAD	36 (6.7)	11 (8.0)	25 (6.2)	0.455
Stroke	59 (10.9)	18 (13.1)	41 (10.1)	0.332
ICH	25 (4.6)	4 (2.9)	21 (5.2)	0.272
Smoke, *n* (%)	193 (35.7)	49 (35.8)	144 (35.6)	0.979
Alcohol, *n* (%)	136 (25.2)	37 (27.0)	99 (24.6)	0.570
Antiplatelet, *n* (%)	38 (7.0)	10 (7.4)	28 (6.9)	0.868
Antihypertensive, *n* (%)	163 (30.1)	47 (34.3)	116 (28.7)	0.218
GCS, median (IQR)	15.0 (14.0, 15.0)	15.0 (13.0, 15.0)	15.0 (14.0, 15.0)	0.095
Initial NIHSS, median (IQR)	2.0 (0, 6.0)	2.0 (0, 6.3)	2.0 (0, 6.0)	0.762
SBP, mmHg	150.0 (136.0, 168.0)	150.5 (139.5, 170.0)	150.0 (136.0, 168.0)	0.235
DBP, mmHg	86.0 (78.0, 96.0)	87.5 (78.0, 96.3)	86.0 (79.0, 96.0)	0.989
MAP, mmHg	108.0 (98.3, 117.7)	108.3 (98.6, 118.7)	107.7 (98.3, 118.7)	0.501
ICH volume at admission (mL), median (IQR)	8.0 (3.0, 17.3)	10.5 (4.3, 23.8)	7.0 (2.4, 15.4)	0.002
Intraventricular extension, *n* (%)	72 (13.3)	23 (16.8)	49 (12.1)	0.165
Subarachnoid extension, *n* (%)	24 (4.4)	7 (5.1)	17 (4.2)	0.658
Hematoma expansion, *n* (%)	28 (5.2)	9 (6.6)	19 (4.7)	0.394
Location of sICH, *n* (%)				
Lobe	171 (31.6)	53 (38.7)	118 (29.2)	0.039
Deep structure	328 (60.7)	82 (59.9)	246 (61.0)	0.806
Brainstem	30 (5.5)	3 (2.2)	27 (6.7)	0.047
Cerebellum	29 (5.4)	6 (4.4)	23 (5.7)	0.555
WBC, × 10^9^/L	8.5 (6.5, 11.1)	8.9 (6.6, 11.8)	8.4 (6.5, 10.3)	0.188
Hemoglobin, g/L	137.5 (123.0, 151.5)	133.0 (122.0, 156.0)	140.0 (124.0, 151.0)	0.205
Platelet count, × 10^9^/L	197.5 (160.0, 238.5)	199.0 (161.0, 232.0)	197.0 (160.5, 239.5)	0.993
Cholesterol, mmol/L	4.6 ± 1.1	4.7 ± 1.0	4.5 ± 1.1	0.257
LDL, mmol/L	2.8 (2.2, 3.6)	2.7 (2.1, 3.6)	2.9 (2.3, 3.7)	0.906
HDL, mmol/L	1.1 (0.9, 1.3)	1.1 (0.9, 1.4)	1.1 (0.9, 1.3)	0.376
Triglyceride, mmol/L	1.3 (0.9, 1.9)	1.4 (0.9, 2.0)	1.3 (0.9, 1.9)	0.949
AST, U/L	22.0 (18.0, 28.0)	21.0 (17.0, 24.0)	23.0 (19.0, 30.0)	0.810
ALT, U/L	20.0 (14.0, 29.5)	20.0 (14.0, 25.0)	20.0 (14.5, 33.0)	0.127
Fasting glucose, mmol/L	7.0 (6.1, 8.8)	6.9 (5.8, 8.5)	7.1 (6.1, 8.8)	0.833
Creatinine, mmol/L	69.4 (60.0, 83.1)	71.2 (60.9, 81.5)	66.5 (52.4, 85.2)	0.175
Urea, mmol/L	6.1 (4.6, 7.3)	6.2 (5.3, 7.0)	5.8 (4.2, 7.3)	0.006
Fibrinogen, mg/dl	301.6 (249.7, 359.8)	320.0 (253.5, 371.2)	295.1 (247.3, 327.0)	0.003
INR	1.0 (1.0, 1.1)	1.0 (0.9, 1.1)	1.0 (1.0, 1.1)	0.846
D-dimer, mg/L	0.5 (0.2, 1.3)	0.6 (0.3, 1.3)	0.4 (0.2, 1.3)	0.029

Data are represented as number (percentage), mean ± standard deviation, or median (interquartile range). *P* < 0.05 was considered to be statistically significant.

R-DWI, remote diffusion weighted imaging; BMI, body mass index; CAD, coronary artery disease; ICH, intracerebral hemorrhage; GCS, glasgow coma scale; NIHSS, national institutes of health stroke scale; SBP, systolic blood pressure; DBP, diastolic blood pressure; MAP, mean arterial pressures; WBC, white blood cell count; LDL-C, low-density lipoprotein cholesterol; HDL-C, high-density lipoprotein cholesterol; AST, aspartate aminotransferase; ALT, alanine transaminase; INR, international normalized ratio.

**Table 2:** Radiological features between subjects with and without DWI lesions.

Variables	All	DWI(+)	DWI(-)	*p*
	***n*** = **541**	***n*** = **137**	***n*** = **404**	
EPVS				
CSO-EPVS, median (IQR)	1.0 (0, 2.0)	2.0 (0, 3.0)	1.0 (0, 2.0)	< 0.001
Severe CSO-EPVS (score>2), *n* (%)	128 (23.7)	64 (46.7)	64 (15.8)	< 0.001
BG-EPVS, median (IQR)	1.0 (1.0, 2.0)	2.0 (1.0, 3.0)	1.0 (0, 2.0)	< 0.001
Severe BG-EPVS (score>2), *n* (%)	117 (21.6)	55 (40.1)	62 (15.3)	< 0.001
WMH				
PV-WMH, median (IQR)	1.0 (0, 2.0)	2.0 (1.0, 3.0)	1.0 (0, 2.0)	< 0.001
DWMH, median (IQR)	1.0 (0, 2.0)	2.0 (1.0, 2.0)	1.0 (0, 2.0)	< 0.001
Total WMH score, median (IQR)	2.0 (1.0, 4.0)	4.0 (2.0, 5.0)	2.0 (0, 3.0)	< 0.001
Number of lacunes, median (IQR)	1.0 (0, 3.0)	2.0 (1.0, 4.0)	1.0 (1.0, 3.0)	< 0.001
Number of CMB, median (IQR)	0 (0.1.0)	1.0 (0, 2.0)	0 (0, 0)	< 0.001
Total CSVD score, median (IQR)	2.0 (0, 3.0)	3.0 (2.0, 4.0)	1.0 (0, 2.0)	< 0.001
Modified CSVD score, median (IQR)	1.0 (0, 3.0)	3.0 (2.0, 4.0)	1.0 (0, 2.0)	< 0.001
Moderate to severe ICAS stenosis (≥50% stenosis or occlusion), *n* (%)	111 (20.5)	46 (33.6)	65 (16.1)	< 0.001
Internal carotid artery	32 (5.9)	16 (11.7)	16 (4.0)	0.001
Anterior cerebral artery	27 (5.0)	16 (11.7)	11 (2.7)	0.059
Middle cerebral artery	48 (8.9)	21 (15.3)	27 (6.7)	< 0.001
Posterior cerebral artery	27 (5.0)	18 (13.1)	9 (2.2)	0.326
Vertebral artery	19 (3.5)	14 (10.2)	5 (1.2)	0.919
Basilar artery	3 (0.6)	3 (5.1)	0 (0.0)	0.575
Low CSVD burden + no or mild ICAS, *n* (%)	315 (58.2)	46 (33.6)	269 (66.6)	< 0.001
High CSVD burden + no or mild ICAS, *n* (%)	108 (20.0)	41 (29.9)	67 (16.6)	0.001
Low CSVD burden + moderate to severe ICAS, *n* (%)	57 (10.5)	13 (9.5)	44 (10.9)	0.644
High CSVD burden + moderate to severe ICAS, *n* (%)	61 (11.3)	38 (27.7)	23 (5.7)	< 0.001

Data are represented as number (percentage), mean ± standard deviation, or median (interquartile range). *P* < 0.05 was considered to be statistically significant.

EPVS, enlarged perivascular space; BG-EPVS, basal ganglia-EPVS; CSO-EPVS, centrum semiovale-EPVS; WMH, white matter hyperintensity; PV-WMH, periventricular WMH; DWMH, deep WMH; CMB, cerebral microbleed; CSVD, cerebral small vessel disease; ICAS, intracranial atherosclerosis.

Patients with DWI lesions were older (62.6 vs. 56.2, *P* < 0.001), had more diabetes (26.5% vs. 7.6%, *P* = 0.006), and larger ICH volume (10.5 cm^3^ vs. 7.0 cm^3^, *P* = 0.002) than those without. The DWI+ group had higher urea, fibrinogen, and D-dimer levels (all *P* < 0.05). No other differences were noted.

### Association between CSVD and DWI lesions

The five CSVD categories—EPVS, WMH, CMB, and lacune—showed significant differences between groups with and without DWI lesions, as detailed in Table 2. The DWI+ group exhibited higher scores on both the original (3.0 vs. 1.0, *P* < 0.001) and modified (3.0 vs. 1.0, *P* < 0.001) total CSVD scores compared to the DWI— group.

Table 3 summarizes the relationship between CSVD and DWI lesions based on logistic regression analysis. In model 1, we found that BG-EPVS (OR 1.65, 95% CI 1.38–1.97) and CSO-EPVS (OR 1.57, 95% CI 1.34–1.83), periventricular WMH (OR 1.99, 95% CI 1.58–2.52), and deep WMH (OR 1.72, 95% CI 1.40–2.11), as well as total WMH score (OR 1.45, 95% CI 1.28–1.64), number of lacunar infarcts (OR 1.19, 95% CI 1.10–1.29), and CMB (OR 1.09, 95% CI 1.03–1.15), were independently associated with the presence of DWI lesions. After additional adjustments for other vascular risk factors and relevant variables in model 2, these associations between CSVD and DWI remained significant, with all *P*-values less than 0.05.

**Table 3:** Relationships between imaging markers of CSVD and DWI lesions.

Variables	Model 1		Model 2	
	**Adjusted OR (95% CI)**	***p***	**Adjusted OR (95% CI)**	***p***
CSO-EPVS score	1.57 (1.34–1.83)	< 0.001	1.54 (1.31–1.82)	< 0.001
BG-EPVS score	1.65 (1.38–1.97)	< 0.001	1.70 (1.40–2.05)	< 0.001
PV-WMH score	1.99 (1.58–2.52)	< 0.001	2.01 (1.58–2.58)	< 0.001
DWMH score	1.72 (1.40–2.11)	< 0.001	1.73 (1.38–2.16)	< 0.001
Total WMH score	1.45 (1.28–1.64)	< 0.001	1.72 (1.26–2.36)	0.001
Number of Lacunes	1.19 (1.10–1.29)	< 0.001	1.19 (1.09–1.29)	< 0.001
Number of CMBs	1.09 (1.03–1.15)	0.001	1.10 (1.04–1.17)	0.001

Model 1: adjusted for age and sex.

Model 2: model 1 + BMI, ICH volume, diabetes, hypertension, urea, fibrinogen, D-dimer, SBP, MAP, NIHSS.

For analyzing CSVD burden, both the original (OR 2.14, 95% CI 1.75–2.63) and modified (OR 1.95, 95% CI 1.65–2.31) total CSVD scores show associations with DWI lesions, with no apparent difference between them. Even after excluding the influence of ICAS factors in model 3, the correlation remained significant (Table 4).

**Table 4:** Relationships between imaging markers of CSVD, ICAS, and DWI lesions.

Variables	Model 1		Model 2		Model 3	
	**Adjusted OR (95% CI)**	***p***	**Adjusted OR (95% CI)**	***p***	**Adjusted OR (95%CI)**	***p***
Total CSVD score	2.08 (1.73–2.51)	< 0.001	2.14 (1.75–2.63)	< 0.001	2.08 (1.69–2.55)	< 0.001^a^
Modified CSVD score	1.87 (1.60–2.18)	< 0.001	1.95 (1.65–2.31)	< 0.001	1.91 (1.61–2.26)	< 0.001^a^
Number of moderate to severe ICAS	1.55 (1.23–1.96)	< 0.001	1.56 (1.22–1.99)	< 0.001	1.54 (1.25–2.00)	0.003^b^
Presence of moderate to severe ICAS	2.20 (1.40–3.48)	0.001	2.28 (1.41–3.68)	0.001	2.27 (1.39–3.77)	0.019^b^

Model 1: adjusted for age and sex.

Model 2: model 1 + BMI, ICH volume, diabetes, hypertension, urea, fibrinogen, D-dimer, SBP, MAP, NIHSS.

Model 3: model 2 + a ICAS or total b CSVD score.

### Association between ICAS and DWI lesions

ICAS was present in 111 (20.5%) of all ICH patients, including 32 (5.9%) in the ICA, 27 (5.0%) in ACA, 48 (8.9%) in MCA, 27 (5.0%) in PCA, 19 (3.5%) in the vertebral artery, and 3 (0.6%) in the basilar artery. The distribution between ICAS and DWI showed that DWI lesions were more commonly seen in the same hemisphere as ICAS, with 22 (16.0%), compared to 8 (6%) in the opposite hemisphere, *p* = 0.001.

Compared to patients in the DWI- group, the prevalence of moderate to severe ICAS (33.6 vs. 16.1%) was higher in the DWI+ group. In the analysis of different ICAS, ICA and MCA were significantly different between the two groups. In model 2, multivariable analyses showed that ICAS was associated with DWI lesions (OR 2.28; 95% CI 1.41–3.68; *P* = 0.001), and this association remained significant after further adjustment for the score of the original CSVD score (OR 2.27; 95% CI 1.39–3.77), as shown in Table 4.

### Association between coexisting ICAS and CSVD and DWI lesions

Among the DWI+ group, 46 (33.6%) patients have a low CSVD burden with no or mild ICAS, 41 (29.9%) have a high CSVD burden with no or mild ICAS, 13 (9.5%) have a low CSVD burden with moderate to severe ICAS, and 38 (27.7%) have a high CSVD burden with moderate to severe ICAS. The difference between the DWI+ and DWI- groups was significant, except for the group with low CSVD burden and moderate to severe ICAS.

**Figure 2:**
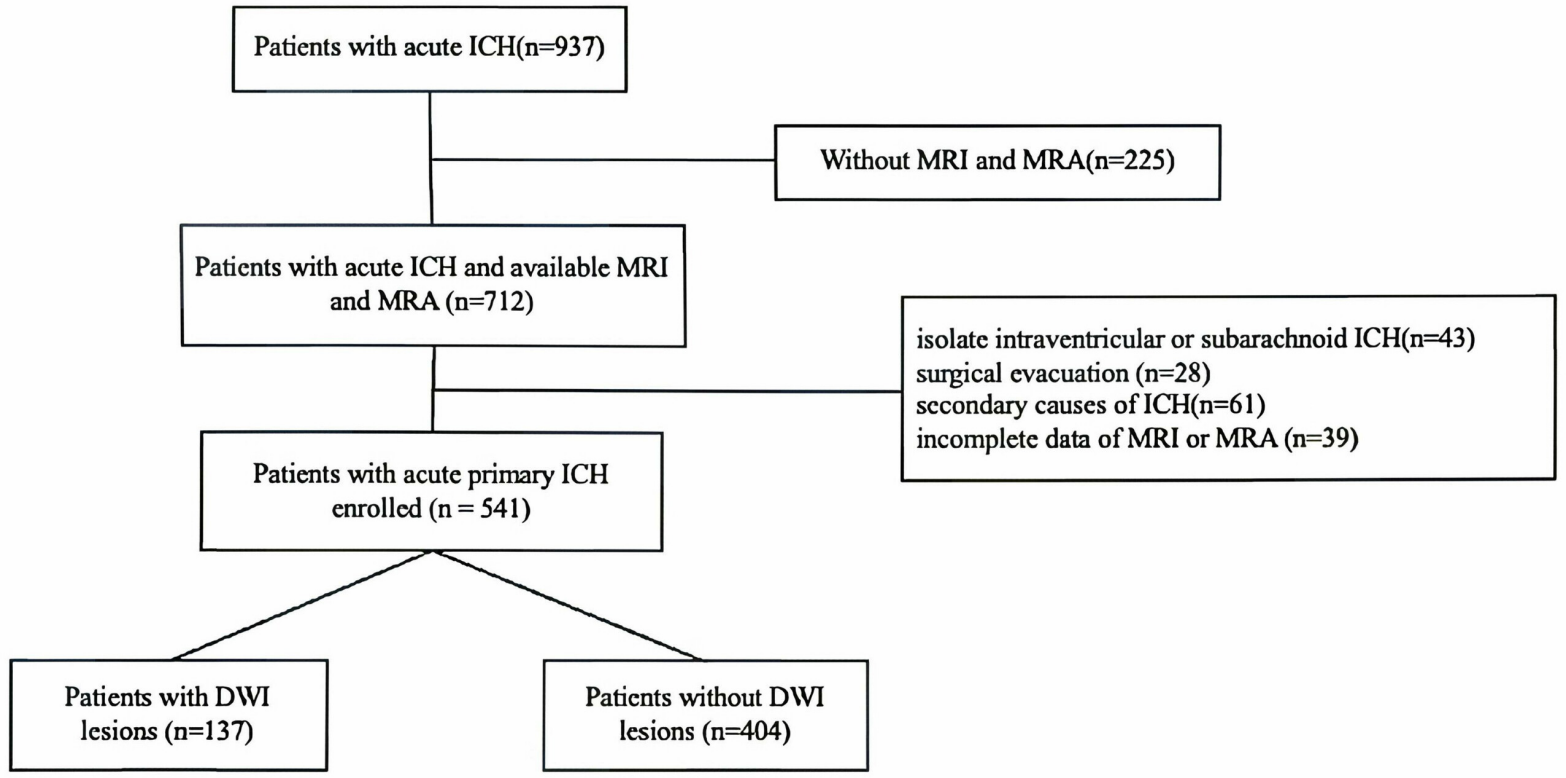
Relationship between severity of ICAS, total CSVD score and DWI lesions.

Multiple logistic regression analysis showed that patients in the high CSVD burden with no or mild ICAS group (OR 3.947; 95% CI 1.824–8.543) and those in the high CSVD burden with moderate to severe ICAS group (OR 4.774; 95% CI 2.088–1.917) were significantly associated with the presence of DWI after adjusting for age and sex. After further adjustment for other relevant factors, the associations remained significant, and the OR was higher in the high CSVD burden with moderate to severe ICAS group than in the high CSVD burden with no or mild ICAS group, as shown in Figure 2.

The horizontal axis is plotted on a logarithmic (base 10) scale. The null value (OR = 1) is positioned at the midpoint. Distances from 0.1 to 1 and from 1 to 10 are equal, and similarly from 0.5 to 1 equals from 1 to 2, ensuring symmetric representation of effect estimates above and below 1. Tick labels show original OR values.

## Discussion

This study found that both CSVD markers detected by brain MRI and moderate to severe ICAS were linked to DWI lesions in ICH patients, respectively. Additionally, our main finding is that the combined burden of CSVD and ICAS, rather than either alone, is most strongly associated with acute DWI lesions after ICH. This suggests that patients with coexisting conditions might have a higher risk of DWI lesions compared to those with a single disease.

Previous population-based studies have explored the relationships between individual CSVD markers such as WMH (4, 6, 8), CMB (3, 15), and CSO-EPVS (24), and DWI lesions in ICH patients. However, the connection between lacunar and BG-EPVS and DWI lesions remains controversial. Compared to CSO-EPVS, BG-EPVS are a stronger marker of hypertensive angiopathy, showing greater correlations with periventricular and subcortical WMH and a closer association with ischemic stroke (17, 25–27). Perivascular spaces (PVS) are fluid-filled cavities surrounding small penetrating cerebral arterioles and venules responsible for draining waste from the brain. Disruption of PVS can lead to the accumulation of toxic metabolic products, damaging the brain's microenvironment and causing widespread EPVS, particularly BG-EPVS. This widespread involvement of EPVS can affect the entire brain and may result in DWI lesions. Lacune can also lead to endothelial dysfunction and blood-brain barrier disruption, which may promote the development of atherosclerosis and result in DWI lesions (28).

Apart from total CSVD burden, a previous study found a difference in 6-point total CSVD burden between patients with and without DWI lesions, but the associations were not explored (29). The Zhejiang study showed that total CSVD burden was independently linked to DWI lesions, using either a 4- or 6-point scale (10). In our study, we obtained similar results and found that a high-grade modified CSVD score better predicts DWI lesions. We believe that the modified CSVD score offers a more precise distinction of WMH, BG-EPVS, and CMB in terms of severity and location, which may more effectively reflect the overall impact of CSVD.

The impact of large arteries on predicting ischemic stroke and future stroke outcomes in asymptomatic individuals has been extensively shown in previous research (19, 30–32), but studies on the link between large and small cerebral arteries in ICH patients remain limited. One study found that CSVD is associated with small DWI lesions, and ICAS is linked to large DWI lesions in ICH patients (14). In our research, we observed that ICAS was related to small DWI lesions and that the coexistence of large and small arteries with DWI lesions showed a stronger association. Similar to a study from Fudan University, which found that coexisting CSVD was very common among ICAS patients (30), and the coexistence of severe ICAS and high-grade CSVD carried a higher stroke risk than just one condition, as reported from Peking Union Medical College Hospital (32).

This co-occurrence suggests that ICAS may critically reduce cerebrovascular reserve. In a brain already compromised by the diffuse injury of CSVD, the stress of ICH (e.g., blood pressure fluctuations, inflammation) may overwhelm compensatory mechanisms, causing focal ischemia in border zone areas or distant microvascular territories. Prior studies have shown that vulnerable carotid plaques often display severe or advanced atherosclerosis, which can exist in the trunk and the terminal vascular beds (33). The presence of multiple large artery atherosclerosis increases the risk of arterial thromboembolic formation at narrowing points and hampers emboli clearance due to reduced blood flow and perfusion deficits in the affected arteries (34). Additionally, research has shown that carotid vulnerable plaques with coexisting lacunes are more strongly linked to acute ischemic stroke than vulnerable plaques alone in symptomatic patients (35), and the ischemic lesions caused by CSVD are considered to lead to a state of chronic hypoperfusion in the white matter, arteriolosclerosis of small vessels, or microthrombus in cerebral large vessels (36). Furthermore, a previous study found cerebral atherosclerosis may lead to chronic and systemic inflammation, reflecting and enhancing the CSVD and causing ischemic events after ICH (37). Inflammation in the brain has been associated with platelet and coagulation cascade activation, which can lead to the formation of DWI lesions (38).

Some limitations of this study should be considered. First, this study enrolled patients who had an MRI within 14 days of ICH onset; however, patients with unstable hemodynamic status who could not tolerate MRI were excluded, which may introduce some selection bias. Secondly, using MRA to evaluate vascular condition in this study tends to overestimate stenosis and perform poorly in distal vessels, where CTA and DSA might have been better examination methods.

Thirdly, the retrospective, single-center design of this study limits the strength of causal inference, so we could not determine a causal relationship between ICAS or CSVD and the development of DWI lesions in ICH patients. Future prospective, multicenter cohort studies are needed to link these imaging findings with clinical outcomes (e.g., mRS scores) to better understand their ultimate significance for management and to explore their clinical translational value. Fourthly, we only measured the degree of stenosis in patients with ICH; we did not evaluate vascular compensation or cerebral perfusion, which could be useful for understanding the mechanisms involved.

Scientifically, this moves beyond cataloging individual markers and points to a “multi-hit” model of vascular vulnerability. Clinically, identifying this high-risk imaging phenotype could, in the future, help select patients for more intensive monitoring or targeted neuroprotective strategies, pending validation in outcome-based studies.

## Conclusion

This study suggests that both ICAS and CSVD are associated with DWI lesions in ICH patients, and moderate to severe ICAS combined with CSVD may have better predictive value for DWI lesions.

## Data Availability

The data analyzed in this study is subject to the following licenses/restrictions: The data that support the findings of this study are not publicly available due to privacy reasons, but are available from the corresponding author upon request. Requests to access these datasets should be directed to yanglei1228@163.com.
