# Supplementary material for: Associations of intracranial arterial stenosis and cerebral small vessel diseases with acute ischemic lesions in spontaneous intracerebral hemorrhage
Source: Front Neurol. 2026 May 8;17:1810507. doi: 10.3389/fneur.2026.1810507 (PMC13196344; doi:10.3389/fneur.2026.1810507)
Supplement: Supplementary file 1 [file Data_Sheet_1.pdf]

Supplementary Figure:Distribution of DWI lesions and ICAS.

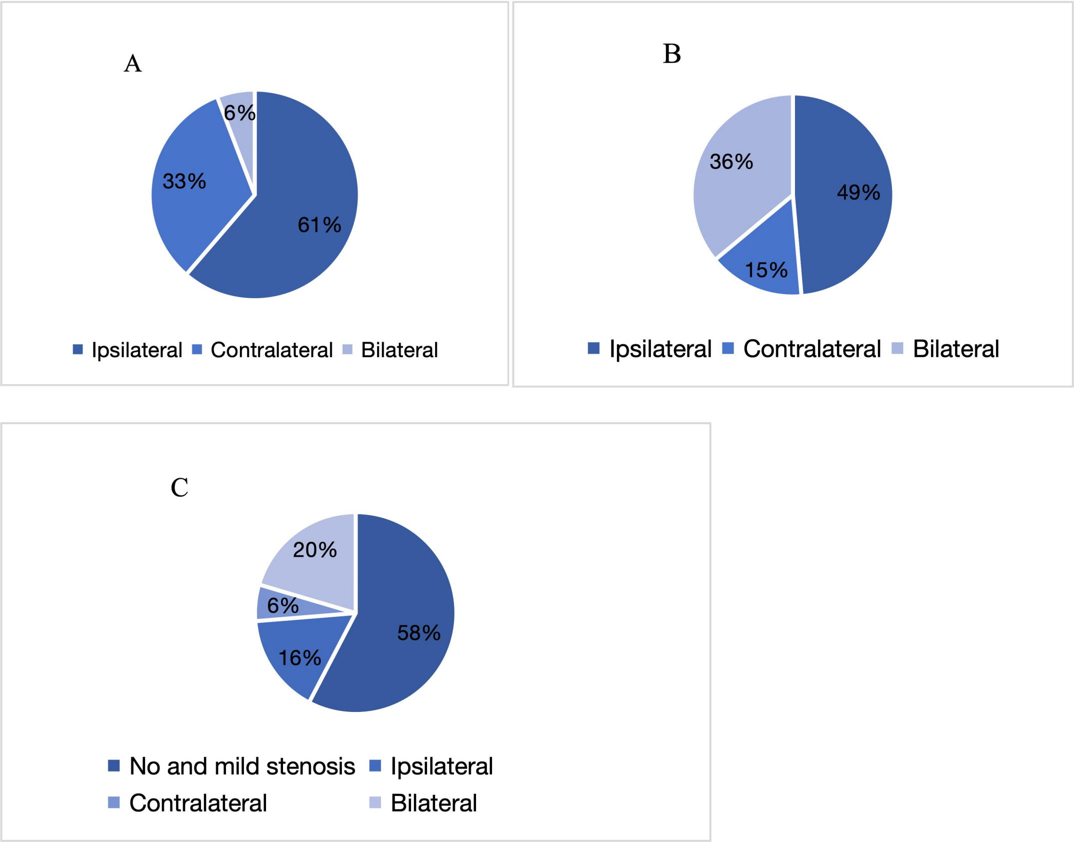

A DWI lesions distribution in different hematoma; B ICAS distribution in different hematoma; C ICAS distribution in DWI lesions;
